# mcIRBP-19 of Bitter Melon Peptide Effectively Regulates Diabetes Mellitus (DM) Patients’ Blood Sugar Levels

**DOI:** 10.3390/nu12051252

**Published:** 2020-04-28

**Authors:** Pang-Kuei Hsu, Frank F. C. Pan, Ching-Sen Hsieh

**Affiliations:** 1 Greenyn Biotechnology Co., Ltd., Taichung City 40768, Taiwan; leon@greenyn.com.tw; 2 Department of Pharmacy and Department of Hospitality, College of Pharmacy and Healthcare, Tajen University, Yanpu, Pingtung County 90741, Taiwan; yongyi.biotech@gmail.com

**Keywords:** bitter melon (*Momordica charantia*), diabetes mellitus, glycated hemoglobin (HbA1c), fasting glucose (FG)

## Abstract

This study was conducted to test the effectiveness of a particular bitter melon peptide (BMP), with a specific sequence of 19 amino acids (mcIRBP-19), in regulating diabetic patients’ blood glucose. In order to test the product with the specially processed BMP, a total of 142 diabetic patients were solicited as study subjects, of which 64 were assigned to an experiment group and 78 to a control group. Biochemical data were compared with a paired *t*-test to verify the significance of changes over different time periods. The clinical results showed that BMP started to improve the subjects’ glycated hemoglobin (HbA1c) levels at the end of the second month (T2), with mean values being significantly lowered from 7.8 ± 1.4% (T0) to 7.5 ± 1.4% (T2) (*p* = 0.004). The values reduced continuously, eventually reaching 7.4 ± 1.1% (*p* = 0.000) at the end of the experiment (T3). HbA1c levels for the control group were 7.5 ± 1.2% in T0 and 7.5 ± 1.1% (T3), and not significantly different (*p* = 0.852) over the same period. This study provides clinical evidence that helps to verify the effectiveness of the new BMP product in regulating diabetic patients’ blood sugar levels.

## 1. Introduction

According to a World Health Organization (WHO) report, the number of people with diabetes mellitus (DM) worldwide has soared from 128 million in 1980 to 422 million in 2014. DM is one of the major threats to human life in many countries. Health-care professionals usually recommend controlling patients’ blood sugar levels through a combination of drugs, diet controls, and regular physical exercise [1,2].

Studies have indicated that a combination of oral medication and insulin injection for 24 weeks is reported to decrease HbA1c by 2.5% [3], but quite a few type-2 diabetic patients refuse to follow this regimen for multiple reasons [4,5].

Bitter melon contains a high dosage of ‘plant insulin’ [6] and can thus effectively lower blood sugar by significantly reducing the speed of nephropathy development in streptozotocin (STZ)-induced diabetic rats [7], improving intestinal disorders caused by diabetes [8], enhancing the function of the intestine, and delaying development of cataracts [9]. Research has further indicated that BMP has a hypoglycemic effect upon insulin-like biological activity and can regulate blood sugar to normal levels [10,11].

The specific sequence of 19 amino acids (mcIRBP-19) extracted from bitter melon as a bioactive peptide may bind insulin receptor (IR) and lower blood glucose, functioning like insulin [12]. This means that BMP has potential as an oral plant insulin for the treatment of diabetes. Many bitter melon products have been developed and it has been claimed that they have hypoglycemic effects. However, none of these products has been clinically tested. The purpose of this study was to test the effect of a product of BMP (produced using an innovative process) clinically on diabetic patients’ blood sugar levels.

## 2. Materials and Methods

### 2.1. Subject

This research was conducted with consideration of human rights that conform to The Universal Declaration of Ethical Principles for Psychologists [13]. The study was approved by the Institutional Review Board (IRB) of PCH (Pingtung Christian Hospital, Pingtung City, Taiwan) with the number IRB552B, prior to approaching prospective subjects.

A total of 142 subjects were included in this study: all were over 20 years old, with independent consciousness and behavior, and were diagnosed by qualified physicians as DM patients. Subjects were patients who collect repeat prescriptions of medications for chronic diabetes from local pharmacies under a government-supported diabetes management program. Sixty-four subjects were randomly assigned to the experimental group and 78 to the control group.

Participants had either a fasting blood glucose (FG) level of at least 140 mg/dL, or an HbA1c of at least 7.0%, and had been diagnosed with diabetes at least one year before the study. Most were issued with a diabetic care manual for continuous care.

### 2.2. Intervention Material

Capsules of BMP (contains mcIRBP-19 with the brand name of Insumate^®^) were obtained from Greenyn Biotechnology Co., Ltd. (Taichung, Taiwan) as per the intervention material. One capsule contains 300 mg of BMP, and the designated dose for this project was 600 mg daily. Therefore, the study subjects were advised to take one capsule before lunch and dinner each day and not to take them within 30 min of other medications. For safety reasons, participants were advised to take their physician’s prescribed hypoglycemic medication as usual, and not to try to replace any of the physician’s prescribed treatments during the study period. In addition, all participants received formal health education on controlling DM, and identical educational material regarding daily diet, regular exercise, and medication adherence. However, such parameters were not controlled in these patients for the present study.

**Figure A1:**
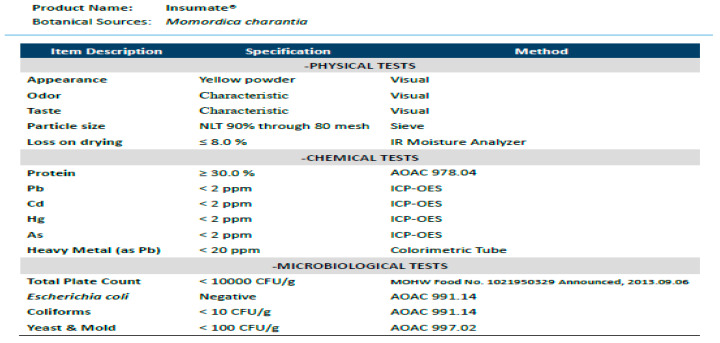
The specifications of Bitter melon peptide. Pb, Plumbum; Cd, Cadmium; Hg, Mercury; As, Arsenic.

The BMP used in this study was a water-extraction powder from bitter melon, containing more than 30% protein and around 1000 ppm mcIRBP-19 peptide, i.e., each capsule of BMP contains around 300 ppm mcIRBP-19 peptide. The specifications and nutritional facts of the BMP product are indicated as Figure A1 and Table A1 in the Appendix A.

Previous studies have reported that the hypoglycemic activity of BMP activates through regulating the insulin receptor [10], and the bioactivity of mcIRBP-19 was confirmed by a further study [12]. In our previous studies in a murine model for a duration of 8 weeks, one study took dosages ranging from 20 mg to 500 mg/day (human equivalent dose), and the other from 250 to 500 mg/day. The levels of fasting glucose (FG) and glycated hemoglobin (HbA1c) started to decrease significantly for the test group in both studies with a dosage of 250 mg/day in the sixth week.

Results from our pilot studies illustrated that a BMP capsule significantly reduces FG and HbA1c starting with a dosage of 250 mg/day and reached optimal outcomes with 500 mg/day. We concluded from these studies that 500 mg/day was the optimal choice. Based on these findings, we adjusted the BMP dose to 600 mg/day for our clinical study of DM2 patients. Each capsule contains 300 mg BMP, and the patients from the experiment group received one capsule 15 min before lunch and dinner.

### 2.3. Measurement

Participants of the experimental group were asked to take a blood biochemical test in a qualified laboratory at one-month intervals. Laboratories in the current study had been approved and included as a contracted laboratory by the National Health Insurance Bureau. To qualify as a contracted laboratory, it is necessary to pass the ability test regulated by the Taiwan Society of Laboratory Medicine (TSLM) and conform to ISO/IEC17043:2010 of the International Laboratory Accreditation Cooperation (ILAC). These requirements are tested on a continuous basis.

Capsules of BMP were administered orally for 3 months. Treatment compliance was measured by the percentage of the trial products consumed. All samples were collected after an overnight fast from all subjects. For biochemical and hematological indices, including FG, HbA1c, and insulin concentration were measured and tested at baseline and 3 months after the initiation of study. We also monitored some safety indicators, such as aspartate aminotransferase (AST), alanine aminotransferase (ALT), creatinine, blood urea nitrogen (BUN), uric acid and hemoglobin (Hb), along with health condition indicators (i.e., blood pressure, heart rate, weight, body mass index (BMI), body fat).

Data gathered from each measurement were analyzed with SPSS 20.0 (IBM Corp., Armonk, NY, sourced from TriStar, Kaohsiung). Statistical analysis techniques applied in the study were the descriptive statistics to examine the demographic distribution of subjects, namely an independent *t*-test analysis to examine whether the average difference is significant between the test and control groups, and a paired *t*-test analysis to check the significant difference for those data from the baseline and the final results. All significant levels are set at *p* ≤ 0.05.

## 3. Results

After the intervention with the capsules of BMP, both experimental and control groups in this study were investigated.

### 3.1. Demographic Characteristics and Concomitant Medication

The subjects in the study were patients who met the diagnostic criteria for diabetes and were under proper medical treatment to control blood sugar. An independent *t*-test analysis revealed that there were no significant differences in the age, body weight, height distributions, blood biochemistry indices (ALT, AST, and creatinine), and the main diabetic indices (FG, HbA1c, and insulin) between the experimental group and the control group. Despite having no significant difference at the beginning, the experimental group members appeared to be older and taller with similar weight than their counterparts in the control group, and both groups were exposed to similar health risks in terms of levels of FG, HbA1c, AST, and ALT with a large standard deviation (Table 1).

### 3.2. BMP Intervention

The blood biochemistry indices in both groups were monitored after 3 months. In Table 2, as a result of paired *t*-testing such that all *p*-values are greater than 0.05, subjects in the control group revealed no significant differences between the first and third monitoring.

The FG result is one of the most used data for blood sugar monitoring in order to judge the performance of diabetes control. Healthy people normally have a 70–110 mg/dL FG level before breakfast. Diabetes could be determined for plasma glucose levels equal to or greater than 126 mg/dL on an empty stomach.

As we expected, the paired *t*-test results of biochemical tests before and after the intervention showed no significant differences in the control group, in which the average fasting blood glucose was 124.9 ± 40.1 mg/dL and 133.2 ± 49.7 mg/dL (*p* = 0.061 > 0.05), and the HbA1c was 7.5 ± 1.2% and 7.5 ± 1.1% (*p* = 0.0852 > 0.05). HbA1c reflected the glycemic control in the human body over 2–3 months. Normally, HbA1c is maintained at 4–6% for a healthy person, and physicians identify a patient as diabetic when the HbA1c reaches 6.50% or above. To ensure quality of life, an HbA1c level of 7% is essential for diabetic patients. Regarding other critical indices, the paired *t*-test results show no significant changes for insulin, triglyceride (TG), total cholesterol (TC), high-density lipoprotein cholesterol (HDL-C), low-density lipoprotein cholesterol (LDL-C), AST, ALT, creatinine, and BUN.

In contrast, significant changes were found in FG and HbA1c for the test group. The paired-*t*-test results in Table 3 show that FG decreased from 136.8 ± 63.5 mg/dL to 118.0 ± 35.5 mg/dL (*p* = 0.007 < 0.05), and HbA1c from 7.8 ± 1.4% to 7.4 ± 1.1% (*p* = 0.000 < 0.05), respectively.

Statistical results of the paired *t*-test, as shown in Table 3, also indicate that some biochemical and hematological indices have changed, e.g., TG, HDL-C, LDL-C, and BUN. The TG of the experimental group decreased from 147.9 ± 85.9 mg/dL to 114.7 ± 64.8 mg/dL (*p* = 0.000 < 0.05) after three months of intervention.

In Table 4, the average FG of the experiment group improved by 5.19% (not statistically significant) from 136.8 ± 64.0 (mg/dL) to 129.7 ± 47.3 by the completion of the first month. The FG of experimental participants reached normal levels, at 120.3 ± 32.3 and 118.0 ± 35.8 for T2 and T3 respectively. The overall improvement was 13.73% from 136.8 ± 64.0 (mg/dL) in T0 to 118.0 ± 35.8 in T3.

Improvements in all periods are mostly statistically significant for T0 − T1 (*p* = 0.165 > 0.05) and T2 − T3 (*p* = 0.539 > 0.05). This means that the intervention material starts to work almost at the beginning of the intervention, and it helps to maintain FG at a rather stable level after T2 (T2 − T3 is not significant). This is especially important in terms of avoiding exposing the patients to a risk of hypoglycemia. In other words, BMP helps to regulate FG to an ideal level, instead of continuously dropping. Notably, the change from T2 to T3 was not statistically significant for the test group; this could be interpreted as the blood level having been regulated to a stable and safe value.

In the same period, levels of FG of the control group increased by 8.13 mg (or 6.89%) from 123.6 ± 42.6 mg/dL in T0 to a higher level of 131.5 ± 51.9 mg/dL in T3 (*p* = 0.155 > 0.05). Although the change was not statistically significant, diabetes control in terms of the FG level was not apparent in the control group.

The HbA1c values give the average glucose levels in human blood, and the HbA1c levels are directly proportional to the blood glucose levels. The right-hand side of Table 4 shows that the average HbA1c of the experimental group drops 0.98% from 7.8 ± 1.4% to 7.7 ± 1.4% in the first month. It decreased further by 3.16% from 7.7 ± 1.4% to 7.5 ± 1.4% at the end of month two, and there was an additional 2.32% improvement from 7.5 ± 1.4% to 7.3 ± 1.5% by the end of month three.

The overall improvement was 0.49 percentage points, or 6.34% in three months. Changes were statistically significant for all periods except for the periods of T0 − T1 and T2 − T3, which means that the capsules of BMP became effective at the start of T2, and the effects remained stable for the rest of the experiment, as shown on the right-hand side of Table 4. After three months of continuous intervention with BMP, the average HbA1c reduced from 7.8 ± 1.4% to 7.3 ± 1.5% (*p* < 0.05). In contrast, the average HbA1c of the control group showed no significant difference during the experimental period (from 7.5 ± 1.2% to 7.5 ± 1.1%, *p* = 0.852 > 0.05).

These results mean that the intervention helped to maintain the levels of blood sugar, which could help patients control diabetes.

We further compared the T3 indices between the test and control groups. It appears that some of them are significantly different, such as TC, TG, and LDL-C, as shown in Table 5. It is interesting to note that the differences in HbA1c, FG, and insulin are not statistically significant.

The values in Table 5 being not significantly different between groups may imply that BMP has no side effects on humans and may be safer to use. Despite that the HbA1c (*p* = 0.300 > 0.05), FG (*p* = 0.80 > 0.05), and insulin (*p* = 0.56 > 0.05) are not significantly different between the experimental and control groups (Table 5), the paired *t*-test of T0 and T3 (Table 2 and Table 3) on same indices are significantly different for the experimental group and not for the control group. This may imply that the BMP is effective, but not significantly superior to the control group for a three-month period. Despite the fact that the experimental group patients can improve their levels of blood glucose with the BMP, the control group may maintain an acceptable level with the prescribed medication.

## 4. Discussion

The subjects were recruited and randomly divided into the experimental group and the control group. The blood biochemical examination showed that there was no statistically significant difference between the experimental group and the control group before the intervention. The age, the history of diabetes, and the blood biochemical examination data were not statistically significantly different. The experimental group received three months of intervention material (the BMP product), while the control group received only health education.

The results showed that the changes in FG and HbA1c are significantly different in the varied period of intervention. On the other hand, the control group showed no improvement in FG and HbA1c. Comparing the changes in these two groups during the same period, the improved range of the experimental group was significantly different from that of the control group.

Bitter melon and its various extracts and components are believed to exert their hypoglycemic effects via different physiological, pharmacological, and biochemical modes [14,15]. The possible modes of the hypoglycemic actions of active ingredients of bitter melon are its hypoglycemic effect [16], stimulation of peripheral and skeletal muscle glucose utilization, inhibition of intestinal glucose uptake, inhibition of adipocyte differentiation, suppression of key gluconeogenic enzymes, stimulation of key enzymes of the hexose monophosphate (HMP) pathway, and preservation of islet β cells and their functions [17].

It was previously reported that bitter melon and its various extracts can stimulate peripheral cell glucose uptake [18]. According to recent studies [11,12], a 68 amino acid sequence peptide from bitter melon exhibited hypoglycemic effects in mice via interaction with the insulin receptor. The studies identified that mcIRBP-19 (50–68, RVRVWVTERGIVARPPTIG) was a putative insulin receptor binding with a b-hairpin structure [11]. The mcIRBP-19 activated the kinase activity of the insulin receptor, stimulated the IR-downstream signaling transduction pathway, enhanced the expression of GLUT4, and increased the glucose uptake in 3T3-L1 cells and the glucose clearance in diabetic mice [10]. Compared with previous animal studies, this clinical study showed that the same changes in pre-meal fasting blood glucose and HbA1c were statistically significantly different within various periods of intervention.

It is noteworthy that, for the test group, TG concentration in plasma was found to be significantly different before and after intervention (Table 3). Similar to the current study, previous studies have also evidenced that bitter melon reduced the adipocyte hyperplasia and had therapeutic potential to prevent or reduce the development of obesity-associated complications [19,20]. One study also suggested that the favorable effects of a bitter melon water extract on increasing insulin sensitivity and attenuating hepatic steatosis may be mediated by enhanced AMP-activated protein kinase (AMPK) and sirtuin (SIRT1) signaling [21].

The literature indicated that patients with diabetes often appear to present with accompanying dyslipidemia symptoms, which include high levels of TG, low HDL-C, and high LDL-C levels. Causes of these symptoms may include insulin resistance [22], and the acceleration of the metabolism of HDL-C when TG is high [23]. The BMP was an insulin-like molecule, which helped to lower blood glucose. After the intervention, the patients’ fasting blood glucose, HbA1c, and TG significantly decreased, as did FG and HbA1c.

As far as the BUN is concerned, high levels of BUN were associated with increased risk of incident diabetes mellitus [24]. The high blood sugar levels damaged millions of nephrons, which jeopardized the kidney’s function of filtrating fluid and maintaining the electrolyte balance [25]. This leads to increased creatinine concentrations in the blood serum. In this study, the results showed that interventional material has some effects on a diabetic patient’s BUN and creatinine, but we may need additional data to prove the relevance of this.

Poor blood sugar control stems from poor diet knowledge or a lack of effective control methods. DM not only causes death through complications but also jeopardizes the patient’s life quality, as well as bringing extra costs to the life of other family members and human society as a whole. The current innovation using BMP could be an additional contribution to the battle against diabetes by improving the level of HbA1c in diabetic patients and helping control blood sugar in a safe way. Although this study showed a significant decrease in blood urea nitrogen and a significant increase in HDL-C (Table 3), this information is not sufficient to prove whether the BMP helps to improve metabolic syndromes or not. In addition, the effect of HbA1c when patients have stopped taking the BMP remains unknown. Future studies should focus on conducting a three-month follow-up to estimate its effect on body function.

## 5. Conclusions

The present study provided clinical evidence to prove that taking 600 mg BMP (Insumate^®^) daily (300 mg each before lunch and supper) can significantly improve the blood glucose of diabetic patients starting from the end of the first month. The findings from this study indicate that the therapeutic benefits that mcIRBP-19-containing capsules have in regulating the blood glucose of diabetic patients may be experienced in a dose-dependent manner. Regulating blood glucose with mcIRBP-19-containing capsules may be safer than other alternatives. However, the BMP should not be used to replace the DM patient’s medication that is prescribed by a physician. The test findings also suggest that a regular intake of BMP may benefit other human organ functions.

## 6. Limitations of the Study

Subjects were taken from two counties with top prevalence rates in Taiwan. The limited number of subjects may not be representative of the entire population of DM patients in Taiwan.

The experiments were conducted over a three-month period. Although the test results are satisfactory and sufficient to prove the effectiveness of the intervention, the current study did not include the changes in HbA1c and other data of the subjects after the end of the intervention. It would be interesting to know how long the effects of such an intervention material might last after a certain period of intervention.

We successfully proved that the intervention material can significantly lower the blood sugar levels and other DM-related levels; however, the mechanism of how this works was not tested in this study.

## Figures and Tables

**Table 1:** Physiological and biochemical indices at the beginning of the study.

Indices	Experiment *n* = 64		Control *n* = 78		*p* ^a^
	Mean	s.d. ^b^	Mean	s.d.	
Age (years)	62.9	12.1	59.3	11.9	0.083
Height (cm)	163.5	6.4	159.7	20.5	0.153
Weight (kg)	69.7	14.1	69.8	16.6	0.803
FG (mg/dL)	136.8	64.0	123.4	42.6	0.138
Insulin (mU/mL)	14.8	17.7	12.1	12.1	0.956
HbA1c (%)	7.8	1.4	7.5	1.2	0.187
Creatinine (mg/dL)	1.3	1.2	2.0	5.7	0.323
AST (U/L)	29.0	13.7	27.0	17.6	0.462
ALT (U/L)	29.7	19.8	26.8	12.8	0.288

^a^ significance level at *p* ≤ 0.05; ^b^ s.d., standard deviation; FG, fasting glucose; HbA1c, glycated hemoglobin levels; AST, aspartate aminotransferase; ALT, alanine aminotransferase.

**Table 2:** Independent *t*-test of biochemical indices for the control group.

Items	Base (*n* = 78)		3-Month (*n* = 78)		*p* ^a^
	Mean	s.d. ^b^	Mean	s.d.	
FG (mg/dL)	123.4	42.6	131.5	51.9	0.155
HbA1c (%)	7.5	1.2	7.5	1.1	0.852
Insulin (mU/mL)	12.1	12.1	14.7	19.4	0.321
TG (mg/dL)	163.0	149.0	165.2	140.3	0.902
TC (mg/dL)	182.2	43.1	181.8	33.6	0.866
HDL-C (mg/dL)	49.2	14.2	48.8	12.3	0.885
LDL-C (mg/dL)	109.4	37.9	108.1	30.0	0.890
AST (U/L)	27.0	17.6	26.3	13.5	0.387
ALT (U/L)	26.8	12.8	28.9	18.7	0.293
Creatinine (mg/dL)	2.0	5.7	4.8	18.8	0.093
BUN (mg/dL)	20.6	8.1	19.7	6.0	0.166

^a^ significance level at *p* ≤ 0.05; ^b^ s.d., standard deviation; TG, triglyceride; TC, total cholesterol; HDL-C, high-density lipoprotein cholesterol; LDL-C, low-density lipoprotein cholesterol; BUN, blood urea nitrogen.

**Table 3:** Independent *t*-test of laboratory values, test group.

Items	Base (*n* = 64)		3-Month (*n* = 64)		*p* ^a^
	Mean	s.d.	Mean	s.d.	
FG (mg/dL)	136.8	63.5	118.0	35.5	0.007
HbA1c (%)	7.8	1.4	7.4	1.1	0.000
Insulin (mU/mL)	14.8	17.6	13.3	11.5	0.345
TG (mg/dL)	147.9	85.9	114.7	64.8	0.000
TC (mg/dL)	165.5	31.6	163.7	27.4	0.554
HDL-C (mg/dL)	46.5	10.5	49.1	12.0	0.050
LDL-C (mg/dL)	96.4	25.9	95.5	25.8	0.676
AST (U/L)	29.0	13.7	29.0	17.2	0.991
ALT (U/L)	29.7	19.8	29.5	18.8	0.881
Creatinine (mg/dL)	1.3	1.2	1.2	0.7	0.100
BUN (mg/dL)	20.5	8.0	19.5	7.0	0.041

^a^ significance level at *p* ≤ 0.05.

**Table 4:** Changes in FG and HbA1c before and after intervention.

Time	FG						HbA1c					
	Mean	s.d.	△ Mean	*t*	*p* ^b^	△%	Mean	s.d.	△ Mean	*t*	*p* ^b^	△%
E ^a^ T0	136.8	64.0					7.8	1.4				
E T1	129.7	47.3	−7.1	1.40	0.165	−5.19	7.7	1.4	−0.1	1.44	0.155	−0.98
E T2	120.3	32.3	−9.4	2.22	0.030	−7.23	7.5	1.4	−0.2	2.32	0.024	−3.16
E T3	118.0	35.8	−2.3	0.62	0.539	−1.92	7.3	1.5	−0.2	1.12	0.269	−2.32
C ^a^ T0	123.6	42.6					7.5	1.2				
C T3	131.5	51.9	8.1	1.44	0.155	6.89	7.5	1.1	0.0	−0.19	0.852	0.36

*n* = 64 (experiment), *n* = 78 (control); ^a^ E for experiment group, C for control group; T0, base time; T1, T2, T3 end of month 1, 2, 3; ^b^ significance level at *p* ≤ 0.05.

**Table 5:** Summary of comparisons between experimental and control groups, T3.

Indices	Experiment	s.d.	Control	s.d.	*t*-test	Sig.
	Mean		Mean		*t*	*p* ^a^
Weight (kg)	66.7	20.3	63.6	27.2	0.76	0.447
BMI	24.7	6.9	24.4	9.6	0.22	0.826
Pulse (bpm)	74.6	19.6	69.4	28.4	1.24	0.217
Body fat (%)	27.7	8.7	28.3	12.6	−0.29	0.769
HbAlc	7.3	1.5	7.5	1.1	−1.04	0.300
FG	118.0	35.8	131.5	51.9	−1.76	0.080
Insulin (mU/mL)	13.0	11.6	14.7	19.4	−0.58	0.560
TC	161.1	34.2	179.5	39.4	−2.97	0.004
TG	112.9	66.4	163.1	141.6	−2.61	0.010
LDL-C	94.0	28.4	106.7	32.4	−2.49	0.014
HDL-C	48.3	13.5	47.5	14.5	0.32	0.746
Creatinine	1.2	0.7	4.8	18.9	−1.54	0.125
AST	29.0	17.2	26.3	13.5	1.01	0.313
ALT	29.5	18.8	28.9	18.7	0.20	0.845
BUN	19.4	7.0	19.0	7.1	0.40	0.690

*n* = 64 (experiment), *n* = 78 (control); ^a^ significance level at *p* ≤ 0.05; BMI, body mass index.

## Appendix A

**Table A1:** Nutritional facts of BMP.

Nutritional Facts per 100 g		
Calories	Kcal/100 g	359.2
Protein	g/100 g	31.1
Total Fat	g/100 g	N. D.
Saturated Fat	g/100 g	N. D.
Trans Fat	g/100 g	N. D.
Total Carbohydrate	g/100 g	58.7
Sugar	g/100 g	N. D.
Water	g/100 g	6.7
Sodium	mg/100 g	4.8

N. D., not detected.
